# Effectiveness and safety of standard chemotherapy in older patients with ovarian cancer: a retrospective analysis by age group and treatment regimen

**DOI:** 10.3389/fonc.2023.1289379

**Published:** 2023-12-13

**Authors:** Miriam R. Brezis, Eliya Shachar, Shira Peleg Hasson, Ido Laskov, Nadav Michaan, Bar Levy, Ido Wolf, Tamar Safra

**Affiliations:** ^1^ Division of Oncology, Tel Aviv Sourasky Medical Center, Tel Aviv, Israel; ^2^ Faculty of Medicine, Tel Aviv University, Tel Aviv, Israel

**Keywords:** older patients, ovarian cancer, chemotherapy, survival, tolerability, toxicity, neoadjuvant chemotherapy

## Abstract

**Objective:**

To evaluate the effectiveness and safety of standard chemotherapy administered to patients >70 years with advanced ovarian cancer (OC).

**Methods:**

Medical records of 956 advanced-stage patients with OC treated between 2002-2020 with standard surgery and paclitaxel-carboplatin chemotherapy in a three-weekly (PC-3W) or weekly (PC-1W) regimen were reviewed. Treatment response and tolerability were compared between patients ≤70 years (N=723) and >70 years (N=233) with stratification to septuagenarians (>70-80 years) and octogenarians (>80 years).

**Results:**

Median overall survival (mOS) in patients >70 was 41.26 months (95% confidence interval [Cl], 37.22-45.14) and median progression-free survival (mPFS) was 11.04 months (95% Cl, 8.97-15.74). No statistically significant differences in mPFS and mOS were observed between septuagenarians and octogenarians. Patients >70 treated with PC-1W versus PC-3W had significantly longer mOS (57.17 versus 30.00 months) and mPFS (19.09 versus 8.15 months). Toxicity rates were mostly similar between younger and older patients. Among patients >70 treated with PC-1W, the rate of neutropenia (75.7% versus 51.8%, p=0.0005), thrombocytopenia (41.0% versus 22.2%, p=0.0042) and anemia (78.1% versus 51.9%, p<0.0001) were significantly higher and the rate of grade 2 alopecia was statistically significantly lower compared with those >70 treated with PC-3W. Significantly more patients treated with PC-1W completed ≥6 chemotherapy cycles, suggesting better tolerability of this regimen.

**Conclusions:**

Older patients with OC may benefit from improved OS with reasonable toxicity if treated with standard chemotherapy. Older patients treated with PC-1W are more likely to complete the full chemotherapy course and survive longer compared with those treated with conventional PC-3W.

## Introduction

About 50% of ovarian cancer cases are diagnosed in women >65 (1). Standard treatment of advanced ovarian cancer includes comprehensive cytoreductive surgery and six cycles of combination therapy with a platinum and taxane with, or without, maintenance bevacizumab and a poly(adenosine diphosphate-ribose) polymerase (PARP) inhibitor. Neoadjuvant chemotherapy with interval debulking surgery is considered in patients with advanced-stage disease who are not good candidates for upfront primary debulking surgery (2). However, less than two-fifths of older women with advanced ovarian cancer received this modified treatment, usually due to poorer performance status or comorbidities (3, 4).

The outcomes of ovarian cancer worsen with age (5, 6). Cumulative analyses from 6 Gynecologic Oncology Group trials estimated that an interval increase of 10 years in age increases the risk for disease progression by 6% and for death by 12% (7). The EUROCARE study showed that relative survival among European women with ovarian cancer was much higher in middle-aged women than in older ones (8).

Several factors make the older population prone to a poorer prognosis, including delayed diagnosis, comorbidities, resistance to chemotherapy, poorer physical and cognitive performance, polypharmacy, depression and frailty (9, 10). Women >65 with advanced ovarian cancer are more likely to have incomplete surgery and more residual disease. These elements were prognostic factors for survival regardless of age (11). In the past, some clinical trials excluded older women, thus suboptimal oncological treatments provided to older patients were based on outcome data from a younger subsection (12).

In a previous analysis we have shown that weekly scheduling of paclitaxel 80 mg/m^2^ and carboplatin area under the curve (AUC) 2, administered on days 1, 8, and 15 in a 28-day cycle (PC-1W) for first-line therapy for advanced ovarian cancer, is as active and better tolerated than the standard regimen of carboplatin and paclitaxel (175 mg/m^2^) every 3 weeks (PC-3W) (13). To challenge the conventional assumption that older patients with advanced ovarian cancer do not tolerate standard therapy, we retrospectively analyzed and compared the effectiveness and tolerability of both regimens (PC-1W and PC-3W) in patients >70 years with advanced ovarian cancer.

## Methods

### Setting and patients

We retrospectively reviewed all medical records of consecutive patients with a diagnosis of advanced (stage III/IV) ovarian cancer, tubal carcinoma, or primary peritoneal carcinomatosis treated at our institution between January 2002 and December 2020. The study was approved by the institutional ethics committee (approval number 07-160). The requirement for informed consent was waived.

Collected data included demographics, background disease, *BRCA* status, mutation type, clinical staging, pathological data, surgical parameters, first treatment line, adverse events during treatment, tumor markers (serum cancer antigen [CA]-125) and survival data. Women with substantial missing treatment outcome data were excluded from the analysis.

### Treatment schedule

Patients underwent primary debulking surgery followed by 6 cycles of carboplatin and paclitaxel, or were treated with 3-4 courses of neoadjuvant chemotherapy followed by interval debulking surgery and complementary 2-3 courses post-surgery. Most patients received 6 chemotherapy cycles. Patients with massive disease received >6 cycles.

Chemotherapy regimens included: conventional doublet paclitaxel 175 mg/m^2^ and carboplatin (AUC 6) every 21 days (PC-3W), or a modified weekly regimen (PC-1W) consisting of paclitaxel 80 mg/m^2^ and carboplatin (AUC 2) administered on days 1, 8, and 15 of a 28-day cycle, or single-agent carboplatin (AUC 6) administered every 21 days.

Treatment protocols remained mostly unchanged for the past 20 years, except for the addition of bevacizumab in 2013 and PARP inhibitors in 2017, following their authorization in Israel.

Treatment regimens were often chosen according to the patient’s performance status. Patients with poorer performance status, older women or women with extremely advanced disease and severe ascites, or those with severe comorbidities were treated with PC-1W. This regimen was also offered to those who preferred having better chances for grade 1 alopecia compared to grade 2 (i.e., complete hair loss). Some patients were treated with carboplatin monotherapy and others refused to receive any chemotherapy.

### Outcomes assessments

Treatment effectiveness was evaluated by computed tomography every 3 chemotherapy cycles. Complete blood count was performed before each treatment. CA-125 levels were assessed once every cycle. A confirmed increase in serum CA-125 levels to more than double the upper limit of normal values (35 U/dL) was considered as evidence for disease progression in patients with no corresponding imaging assessment.

Overall survival (OS) was calculated from the diagnosis date to either death or to the last known follow-up. Progression-free survival (PFS) was calculated from the end of the first platinum-based combination to either progression/recurrence, or death, or to the last known follow-up. Platinum sensitivity was defined as recurrence of disease, after more than 6 months from the end of the first platinum-based treatment.

Toxicity was evaluated according to the National Cancer Institute Common Terminology Criteria for Adverse Events version 2. At each follow-up visit (for the duration of patient follow-up) the patient was also asked if she had long-lasting (>1 year) peripheral sensory neuropathy.

### Statistical analysis

Outcomes were analyzed by patient age: ≤70 years, >70 years, septuagenarians (>70-80), and octogenarians (>80 years). In addition, the outcomes were analyzed by chemotherapy regimen (PC-1W and PC-3W).

Continuous variables were summarized as median and range and compared by independent Student’s t-test. Categorical variables were summarized as number and percentage and compared by Fisher’s exact test or chi-squared test. Survival functions were demonstrated using the Kaplan-Meier method, and the effect of each subgroup was assessed using the Log-Rank test.

The Cox proportional hazards model was used for comparing the risk of death and the risk of a composite endpoint of death or recurrence between the 2 treatment regimens, adjusting for age, stage of disease, histology, *BRCA* status (carriers versus noncarriers), and debulking status. Results are presented as hazard ratio (HR) and 95% confidence interval (CI). All analyses were 2-tailed, and a p value of <0.05 was considered significant. Statistical analyses were performed using R software, version 4.05 (R Development Core Team, Vienna, Austria).

## Results

### Clinical characteristics of the study population

In total, 956 women with stage III/IV ovarian cancer were diagnosed and treated at our institution during the study period. They comprised 87.35% of all patients; the remaining 12.65% had stage I/II disease. As shown in **Table 1**, most patients (723, 75.62%) were >70 at diagnosis. About two-thirds had serous papillary histology (69.87% in patients ≤70 and 67.29% in patients >70; p=0.5893). Optimal cytoreduction (R0) was performed in 65.54% of patients ≤70 versus 51.76% of patients >70 (p=0.0016). Suboptimal cytoreduction (R1 and R2) was significantly more common in older patients. Approximately 70% of patients were tested for *BRCA* mutations; *BRCA* mutations were less common in patients >70. Older patients had significantly higher rates of hypertension and hypercholesterolemia than those ≤70 (p<0.0001).

**Table 1:** Patient demographics and baseline characteristics.

Variables	≤70 N = 723	>70 N = 233	P Value
**Age at Diagnosis**	58 (26-70)	76 (71-91)	<0.0001
Stage			
Stage III	512 (82.58%)	159 (73.61%)	<0.0001
Stage IV	108 (17.42%)	57 (26.39%)	
**Interval debulking**	314 (51.56%)	135 (63.68%)	0.0029
Residual disease			
R0	388 (65.54%)	103 (51.76%)	0.0016
R1	150 (25.34%)	66 (33.17%)	
R2	54 (9.12%)	30 (15.08%)	
Histology			
Serous papillary	429 (69.87%)	144 (67.29%)	0.5893
Adenocarcinoma	172 (28.01%)	64 (29.91%)	
Mucinous, clear cell, carcinosarcoma	13 (2.12%)	6 (2.79%)	
***BRCA* status** (tested)	544 (75.24%)	158 (67.81%)	0.4487
*BRCA1*	156 (21.57%)	23 (3.87%)	0.0005
*BRCA2*	60 (8.29%)	13 (5.57%)	
Negative	328 (45.36%)	122 (52.36%)	
Molecular Testing^*^	179 (23.58%)	57 (24.05%)	0.9521
Medical history^**^			
Hypertension	63 (10.14%)	63 (29.03%)	<0.0001
Diabetes mellitus	38 (6.12%)	18 (8.26%)	0.3436
Hypercholesterolemia	53 (8.53%)	46 (21.2%)	<0.0001
Thyroid disorders	28 (4.51%)	16 (7.37%)	0.1799
Treatment protocol			
PC-3W	360 (60.1%)	99 (45.2%)	<0.0001
PC-1W	233 (38.89%)	93 (42.46%)	
Carboplatin alone	4 (0.66%)	18 (8.21%)	
No treatment	12 (2%)	9 (4.1%)	
Number of courses completed			
<6	56 (9.39%)	35 (16.75%)	0.0316
6	407 (68.29%)	128 (61.24%)	
>7	133 (22.32%)	46 (22.01%)	
Bevacizumab treatment			
First line/After recurrence	127 (20.42%)/ 63 (10.13%)	35 (16.06%)/ 15 (6.88%)	0.0848
PARPi treatment			
First line/After recurrence	28 (4.5%)/ 71 (11.41%)	10 (4.59%)/ 5 (2.29%)	0.0003

*Molecular Testing includes the following exams – FoundationOne, Cacner Hope, HRD, Paradigm, Guardant, Tempus, Invitae, GPS, Topacio, Oncotest, MSKCC, Counsyl.

**Medical history was collected systematically from 2015, thus information was available for 553 patients in the control group and 164 in the older group. PC-1W = neoadjuvant carboplatin (AUC 2) and paclitaxel (80 mg/m^2^) administered on days 1,8 and 15 in a 28-day cycle. Repeated for 6 cycles; PC-3W= neoadjuvant carboplatin (AUC 6) with paclitaxel (175 mg/m^2^) on day 1 of a 21-day cycle repeated for 6 cycles.

Neoadjuvant chemotherapy was given to 51.56% and 63.68% of patients ≤70 and >70 patients, respectively (p=0.0029). Most patients were treated with ≥6 cycles (90.61% of patients ≤70 patients and 83.25% of patients >70, p=0.0316).

The standard PC-3W regimen was given to 60.1% and 45.2% of patients ≤70 and >70, respectively, (p<0.0001). The PC-1W regimen was given to 38.89% and 42.46% of patients ≤70 and >70, respectively (p<0.0001). Carboplatin alone was administered to 0.66% and 8.21% of patients ≤70 and >70, respectively (p<0.0001).

Bevacizumab as first-line therapy and maintenance treatment was administered to 30.55% of patients ≤70 and 22.94% of patients >70, (p=0.0848). PARP inhibitors were administered as first-line therapy to 4.5% of patients ≤70 patients and 4.59% of patients >70. After recurrence PARP inhibitors were administered to 11.41% of patients ≤70 and 2.3% of patients >70 (p=0.0003).

### Survival of patients >70 versus ≤70

**Figure 1:**
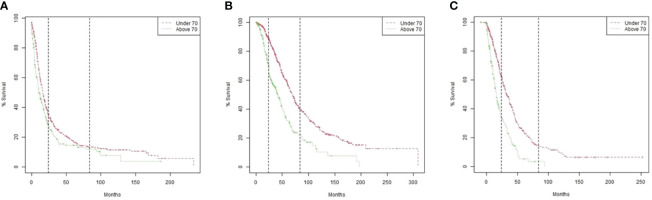
Kaplan Meier Survival models by age group (>70 versus ≤70). **(A)** Overall survival. P value by Log-rank test <0.0001. **(B)** Progression-free survival. P value by Log-rank test 0.0041. **(C)** Overall survival after recurrence according to the time interval from initiation of treatment after the first recurrence to death. P value by Log-rank test <0.0001.

Median PFS, OS, and OS after recurrence were all statistically significantly longer in patients ≤70 compared to those >70 (**Table 2** and **Figure 1**).

**Table 2:** Comparison of treatment response and survival by age.

Survival Data	≤70 N = 723	>70 N = 233	P Value
Median OS, months (95% CI)	69.78 (63.87-74.55)	41.26 (33.05-50.63)	<0.0001
Median PFS, months (95% CI)	16.03 (14.72-18.17)	11.04 (8.97-15.74)	0.0041
Median OS after recurrence, months (95% CI)	32.43 (29.31-37.98)	15.61 (12.75-20.37)	<0.0001
Platinum sensitivity, n (%)	582 (84.84%)	130 (64.36%)	<0.0001

CI, confidence interval; OS, overall survival; PFS, Progression-free survival.

### Comparison of outcomes by chemotherapy regimens administered to patients >70

The effectiveness and tolerability of the PC-1W and PC-3W regimens were compared in patients >70. Age, stage at diagnosis, histological type, *BRCA* status and optimal cytoreduction were similar in both treatment groups (**Table 3**). A higher rate of patients treated with PC-3W versus PC-1W received 1-5 courses (17.93% versus 6.16%, p=0.0103), mainly due to toxicity/intolerance to chemotherapy. Additionally, a lower rate of patients treated with PC-1W versus PC-3W received ≥7 cycles (16.04% versus 29.9%, p=0.0103), mainly due to advanced disease, stage IV or ascites.

**Table 3:** Comparison of older (>70) patients’ characteristics by treatment regimen.

Variables	PC-1W N = 99	PC-3W N=108	P Value
**Age at Diagnosis**	75 (70-86)	74 (70-87)	0.101
Stage			
Stage III	75 (74.26%)	88 (80.00%)	0.4067
Stage IV	26 (25.74%)	22 (20.00%)	
**Interval Debulking**	76 (76.77%)	63 (57.8%)	0.0059
Residual disease			
R0	43 (45.26%)	62 (59.05%)	0.1493
R1	36 (37.89%)	30 (28.57%)	
R2	16 (16.84%)	13 (12.38%)	
Histology			
Serous Papillary	75 (70.75%)	70 (63.06%)	0.2698
Endometrioid	31 (29.24%)	35 (31.53%)	
***BRCA* status (tested)**	78 (78.78%)	80 (74.07%)	
*BRCA1*	11 (11.11%)	10 (9.25%)	0.7229
*BRCA2*	6 (6.06%)	8 (7.40%)	
Negative	61 (61.61%)	62 (57.4%)	
Number of Courses			
1-5	6 (6.18%)	19 (17.93%)	0.0103
6	62 (63.92%)	70 (66.04%)	
7 or more	29 (29.9%)	17 (16.04%)	

PC-1W = neoadjuvant carboplatin (AUC 2) and paclitaxel (80 mg/m2) administered on days 1, 8 and 15 in a 28-day cycle. Repeated for 6 cycles; PC-3W= neoadjuvant carboplatin (AUC 6) with paclitaxel (175 mg/m2) on day 1 of a 21-day cycle repeated for 6 cycles.

Patients treated with PC-1W versus PC-3W showed statistically significant longer median PFS (19.09 months [95% CI 14.55-23.85] versus 8.15 months [95% CI 5.85-10.05], p=0.001) and OS (57.17 months [95% CI 47.74-69.22] versus 30.00 months [95% CI 25.00-45.14], p=0.0092), (**Table 4**). A higher rate of patients treated with PC-1W had platinum-sensitive disease compared to those treated with PC-3W (77.78 versus 56.70%, p=0.0028).

**Table 4:** Comparison of treatment response and survival by treatment regimen in patients >70.

Survival Data	PC-1W N = 99	PC-3W N=108	P Value
Median OS, months (95% CI)	57.17 (47.74-69.22)	30.00 (25.00-45.14)	0.0092
Median PFS, months (95% CI)	19.09 (14.55-23.85)	8.15 (5.85-10.05)	0.001
Platinum Sensitivity, n (%)	77 (77.78%)	55 (56.71%)	0.0028

CI, confidence interval; OS, overall survival; PFS, Progression free survival. PC-1W = neoadjuvant carboplatin (AUC 2) and paclitaxel (80 mg/m^2^) administered on days 1,8 and 15 in a 28-day cycle. Repeated for 6 cycles; PC-3W= neoadjuvant carboplatin (AUC 6) with paclitaxel (175 mg/m^2^) on day 1 of a 21-day cycle repeated for 6 cycles.

### Comparison of treatment characteristics and outcomes between septuagenarians and octogenarians

Stage at diagnosis, histological subtype, induction, optimal cytoreduction, *BRCA* status, and platinum sensitivity were similar in septuagenarians (n=187) versus octogenarians (n=46), (**Table 5**).

**Table 5:** Comparison of older patients’ characteristics by age group (70-80 versus 80 and above).

Variables	70-80 years N = 187	≥80 years N = 46	P Value
**Age at Diagnosis**	74 (70-80)	83 (81-93)	<0.0001
Stage			
Stage III	145 (75.92%)	31 (67.39%)	0.3176
Stage IV	46 (24.08%)	15 (32.61%)	
**Interval debulking**	126 (67.38%)	23 (53.49%)	0.1229
Residual disease			
R0	93 (52.25%)	19 (46.5%)	0.1851
R1	61 (24.27%)	11 (27.5%)	
R2	24 (13.48%)	10 (25%)	
Histology			
Serous Papillary	128 (68.09%)	29 (64.44%)	0.536
Adenocarcinoma, mucinous, clear cell, carcinosarcoma	55 (29.25%)	15 (34.33%)	
*BRCA* status			
*BRCA-1*	21 (11.22%)	3 (6.52%)	0.4768
*BRCA-2*	14 (7.48%)	2 (4.34%)	
Negative	107 (57.21%)	26 (56.52%)	
Chemotherapy protocol			
PC-1W	80 (41.88%)	21 (43.75%)	<0.0001
PC-3W	98 (51.3%)	12 (25%)	
Carboplatin alone	8 (4.81%)	10 (20.83%)	
Number of Courses			
1-5	25 (13.59%)	12 (27.27%)	0.0203
6	114 (61.96%)	27 (61.36%)	
7 or more	45 (24.46%)	5 (11.36%)	
Platinum Sensitivity	121 (67.6%)	25 (58.14%)	0.3199

PC-1W = neoadjuvant carboplatin (AUC 2) and paclitaxel (80 mg/m^2^) administered on days 1, 8 and 15 in a 28-day cycle. Repeated for 6 cycles; PC-3W= neoadjuvant carboplatin (AUC 6) with paclitaxel (175 mg/m^2^) on day 1 of a 21-day cycle repeated for 6 cycles.

PC-1W and single-agent carboplatin were more frequently administered to octogenarians compared to septuagenarians (p<0.0001). A higher percentage of octogenarians received 1-5 courses compared to septuagenarians (p<0.0001).

A trend for statistically significant longer median OS was observed in septuagenarians versus octogenarians (47.74 months [95% CI, 38.77-55.29] versus 37.22 months [95% CI, 24.61-65.35], p=0.0792). No statistically significant difference in median PFS was observed between the two age groups (12.45 months [95% CI, 9.82-17.91] versus 9.13 months [95% CI, 5.32-20.30], p=0.3746).

### Toxicity with stratification by age

To evaluate the safety of chemotherapy protocols in older patients, we compared toxicities among all age groups (≤70, >70-80, >80). Only the rates of asthenia were statistically significantly different among the groups (**Table 6**).

**Table 6:** Comparison of toxicities by age group.

Toxicities	≤70 years N=723		>70-80 years N = 187		>80 years N=46		P Value
	Grade 1/2	Grade 3/4	Grade 1/2	Grade 3/4	Grade 1/2	Grade 3/4	
Neutropenia	310 (42.9%)	58 (8.0%)	75 (40.1%)	27 (14.4%)	23 (50.0%)	0	0.5467
Thrombocytopenia	139 (19.2%)	44 (6.1%)	41 (21.9%)	11 (5.9%)	9 (19.6%)	3 (6.5%)	0.7848
Anemia	310 (42.9%)	40 (5.5%)	87 (46.5%)	15 (8.0%)	25 (54.3%)	2 (4.4%)	0.1602
Alopecia	478 (66.1%)	7 (0.97%)	117 (62.6%)	4 (2.1%)	30 (65.2%)	0	0.0662
Asthenia	147 (20.3%)	21 (2.9%)	40 (21.4%)	13 (7.0%)	15 (32.6%)	3 (6.5%)	0.0272
Nausea	87 (12.0%)	3 (0.4%)	27 (14.4%)	2 (1.1%)	9 (19.6%)	1 (2.2%)	0.2015
Neuropathy (short term)	278 (38.5%)	23 (3.2%)	68 (36.4%)	8 (4.3%)	13 (28.3%)	0	0.1827
Long-term neuropathy *	172 (23.8%)	2 (0.28%)	48 (25.7%)	0	8 (17.4%)	0	0.5005

*neuropathy>1 year.

Comparison of toxicities between patients treated with PC-1W and PC-3W (**Table 7**) showed a statistically significant higher rate of neutropenia (75.7% versus 51.8%, p=0.0005), thrombocytopenia (41.0% versus 22.2%, p=0.0042) and anemia (78.1% versus 51.9%, p<0.0001) in patients treated with PC-1W versus PC-3W. The rates of all-grade alopecia were similar between the 2 regimens, but the rate of grade 2 alopecia was statistically significantly lower among patients treated with PC-1W compared to PC-3W (26.3% versus 63.9%, p<0.0001). The rate of asthenia was also statistically significantly higher in patients treated with PC-1W compared to PC-3W (39.4% versus 15.7%, p-0.0022). The rates of short-term and long-term neuropathy were similar in both treatment regimens. The rates of grade 3 or higher toxicities were similar in both treatment regimens, except for neutropenia, which was statistically significantly higher in patients treated with PC-1W versus PC-3W (23.2% versus 7.4%, p=0.0017). Two women died within 30 days of surgery. No other records regarding morbidity or mortality after surgery were documented.

**Table 7:** Comparison of toxicities by treatment regimen (PC-1W versus PC-3W) in patients >70.

Toxicities	PC-1W N=99		PC-3W N=108		P Value	
	Grade 1/2	Grade 3/4	Grade 1/2	Grade 3/4	All grades	Grade 3/4
Neutropenia	52 (52.5%)	23 (23.2%)	48 (44.4%)	8 (7.4%)	0.0005	0.0017
Thrombocytopenia	33 (33.3%)	8 (7.7%)	17 (15.7%)	7 (6.5%)	0.0042	0.7901
Anemia	70 (70.0%)	8 (8.1%)	50 (46.3%)	6 (5.6%)	<0.0001	0.5829
Alopecia	79 (79.8%)	1 (1.0%)	78 (72.2%)	4 (3.7%)	0.3148	0.3713
Asthenia	39 (39.4%)	6 (6.1%)	17 (15.7%)	10 (9.3%)	0.0022	0.4435
Nausea	23 (23.2%)	0	15 (13.9%)	2 (1.9%)	0.2176	0.4985
Neuropathy (short term)	41 (41.1%)	2 (2.0%)	50 (46.3%)	7 (6.5%)	0.2106	0.1739
Neuropathy (long term)*	28 (28.3%)	–	41 (38.0%)	–	0.1839	–

PC-1W = neoadjuvant carboplatin (AUC 2) and paclitaxel (80 mg/m^2^) administered on days 1,8 and 15 in a 28-day cycle. Repeated for 6 cycles; PC-3W= neoadjuvant carboplatin (AUC6) with paclitaxel (175 mg/m^2^) on day 1 of a 21-day cycle repeated for 80 mg/m^2^ 6 cycles.

*neuropathy>1 year.

## Discussion

This retrospective study showed that patients >70 treated with the modified PC-1W regimen had significantly longer median OS (57.17 versus 30.00 months) and PFS (19.09 versus 8.15 months) compared to patients >70 treated with the standard PC-3W regimen. The weekly protocol was more tolerable, as reflected by the higher rate of patients who completed ≥6 cycles of treatment compared to those treated with PC-3W, with similar rates of grade 3 toxicities, except for neutropenia. No statistically significant differences in median PFS and median OS were observed between septuagenarians and octogenarians. Toxicity rates were mostly similar between younger and older patients.

Patients >70 had significantly higher rates of comorbidities and advanced disease (stage IV) compared to younger patients, thus, neoadjuvant chemotherapy was more common in older patients. Neoadjuvant chemotherapy with interval debulking surgery is associated with higher rates of optimal cytoreduction, lower perioperative morbidity and mortality rates and similar outcomes, compared to primary debulking surgery (14, 15).

Although there was no difference in *BRCA* testing frequency in the younger and older groups, the rate of *BRCA1/2* mutations was significantly lower in the older population. This may be explained by diagnosis of *BRCA* cancers at a younger age, or an active medical follow-up in patients carrying *BRCA1/2* mutations.

Median PFS and median OS were 11.04 months and 41.26 months, respectively, among patients >70 treated with a platinum-taxane doublet. Liontos et al. (16) reported similar findings with a median PFS of 11.3 months and a median OS of 30.2 months among patients >70 years treated with a platinum-taxane doublet (84% of patients) or carboplatin monotherapy (16%). Although these values are significantly shorter than for younger patients, they suggested that older patients can still benefit from substantial lengthening of their lives.

In a previous study, we have found significantly longer median PFS and OS in patients with ovarian cancer (all stages) treated with PC-1W compared to PC-3W with a survival HR of 0.54 (95% CI, 0.43–0.67; p<0.001) (13). Our current analysis corroborates this finding in older patients. The MITO-7 study showed that carboplatin and paclitaxel given once a week versus every 3 weeks did not significantly prolong PFS in patients >70 years versus ≤70 years but it was associated with better quality of life and fewer toxic effects (17). The EWOC-1 trial provided evidence that the paclitaxel-carboplatin combination should remain the standard of care even for older and frailer patients with ovarian cancer because survival was significantly worse in the carboplatin monotherapy arm (18). In a pan-cancer meta-analysis of 19 randomized clinical trials that included 5 ovarian cancer trials, 6 breast cancer trials, 6 non-small cell lung cancer trials, 1 head and neck squamous cell carcinoma trial and 1 cervical cancer trial, the weekly paclitaxel regimen showed statistically significant better PFS than the 3-week paclitaxel regimen, with a HR of 0.90 (95% CI 0.82–0.99, p=0.02), but no significant difference in OS was found between the 2 paclitaxel regimens. Interestingly, PFS was affected by ethnicity with the weekly paclitaxel regimen showing improved PFS compared to the 3-weekly paclitaxel regimen in North American and Asia, but not in Europe (19).

In contrast to other reports (20), except for higher rates of asthenia in the older population, which is expected, no statistically significantly different rates of toxicities were observed in older patients (>70-80 and >80) compared to younger ones (≤70).

Patients >70 treated with PC-1W had statistically significantly higher hematological toxicities (neutropenia, thrombocytopenia and anemia) as well as asthenia; however, statistically significant difference in grade 3 toxicities was only observed for neutropenia. The higher rate of hematological malignancies in patients treated with PC-1W, may be due to the weekly complete blood count monitoring that these patients undergo prior to receiving weekly treatment, which may lead to the identification of hematological changes that would not have been detected with a longer interval between treatments. In contrast, a lower risk for grade 3/4 toxicities was reported for the weekly paclitaxel schedule compared to the 3-weekly one in the pan-cancer meta-analysis (19). In the current study, no difference in toxicity was observed when comparing septuagenarians to octogenarians, as had been suggested in prior studies (21).

The PC-1W protocol, which is administered over 28 days instead of 21-day cycles, includes a week without treatment, allowing older patients to recuperate. The fact that more patients treated with the PC-1W regimen were able to complete ≥6 cycles of therapy may also be attributed to the 6-week delay in course completion of this regimen (24 weeks instead of 18 weeks), allowing highly frail patients to recover between cycles and increasing the completion rate. Hence, the PC-1W regimen may offer advantages for older patients in terms of tolerance while retaining efficacy, perhaps by allowing more carboplatin to be given.

We further tried to evaluate differences in the characteristics and prognosis of older patients. Treatment guidelines for women >80 are undetermined; some research suggests that octogenarians may not tolerate combined surgery and chemotherapy (22). Octogenarians received more PC-1W in addition to carboplatin monotherapy compared to the septuagenarians. These subgroups had similar median OS and PFS, and no statistically significant differences in toxicities.

The strengths of this study include the assessment of a large patient population in a real-life clinical setting, in addition to a long ~20-year follow-up. The study findings are limited by its retrospective design. Our analysis only included a population of women diagnosed at stage III+IV. Surgical mortality and morbidity were not analyzed in our study. However, only 2 women died within 30 days of surgery in the entire cohort.

This study extends the literature describing treatment of older women with ovarian cancer, demonstrating that chemotherapy may be used with appropriate caution in this population with significantly improved survival rates and moderate toxicities. In recent years it has been suggested that patient frailty should be considered alongside their chronological age when making therapeutic decisions, as these are not synonymous and may allow a considerable number of women to benefit from an optimal treatment (23, 24). While we had no data on patient frailty, we emphasize the importance of increasing the use of a comprehensive geriatric assessment to formally assess the physiological, cognitive, functional and social support that older women receive during treatment planning. Frailer patients may benefit from the milder weekly paclitaxel carboplatin 28-day protocol.

Similar percentages of older patients underwent comprehensive genomic profile testing, but the numbers are too small to evaluate its prognostic contribution. Future studies should evaluate the use of this test in older patients, to improve tailored therapy, particularly in this population. The effects and toxicities of PARP inhibitors and bevacizumab in this population should also be further researched. As the use of PARP inhibitors was only approved for use in Israel for recurrent ovarian cancer in 2017, its use in our cohort was somewhat limited.

## Conclusions

Older women, have substantial prognosis with conservative therapies with tolerable toxicity. Moreover, weekly intermittent therapy with carboplatin and paclitaxel seems less toxic and more effective in the older population and patients are more likely to complete the full chemotherapy course and survive longer. As we rapidly advance our understanding of the response to treatment in older patients, we urge care providers to carefully give them a complete treatment that would offer them an improved probability for longer survival.

## Data availability statement

The raw data supporting the conclusions of this article will be made available by the authors, without undue reservation.

## Ethics statement

The studies involving humans were approved by Tel Aviv Sourasky Medical Center Ethics Committee. The studies were conducted in accordance with the local legislation and institutional requirements. The ethics committee/institutional review board waived the requirement of written informed consent for participation from the participants or the participants’ legal guardians/next of kin because the study was a retrospective review of medical records.

## Author contributions

MB: Data curation, Writing – review & editing. ES: Writing – review & editing. SP: Data curation, Writing – review & editing. IL: Data curation, Writing – review & editing. NM: Data curation, Writing – review & editing. BL: Data curation, Writing – review & editing. IW: Writing – review & editing. TS: Conceptualization, Data curation, Formal Analysis, Writing – original draft, Writing – review & editing.
